# Supplementary material for: The interplay of stiffness and force anisotropies drives embryo elongation
Source: eLife. 2017 Feb 15;6:e23866. doi: 10.7554/eLife.23866 (PMC5371431; doi:10.7554/eLife.23866)
Supplement: Supplementary file 1. — DOI: http://dx.doi.org/10.7554/eLife.23866.018 [file elife-23866-supp1.docx]

| **Sample** | **Number of embryos** | **Sample** | **Number of embryos** |
| --- | --- | --- | --- |
| **H1 1.3F DV** | 20 | ***spc-1(RNAi)* H1 1.7F DV** | 18 |
| **H1 1.3F AP** | 22 | ***spc-1(RNAi)* H1 1.7F AP** | 16 |
| **V3 1.3F DV** | 27 | ***unc-112(RNAi)* H1 1.7F DV** | 26 |
| **V3 1.3F AP** | 22 | ***unc-112(RNAi)* H1 1.7F AP** | 27 |
| **V6 1.3F DV** | 16 | **HYP7-Dorsal 1.3F DV** | 17 |
| **V6 1.3F AP** | 16 | **HYP7-Dorsal 1.3F AP** | 6 |
| **H1 1.5F DV** | 46 | **HYP7-Ventral 1.3F DV** | 18 |
| **H1 1.5F AP** | 47 | **HYP7-Ventral 1.3F AP** | 19 |
| **V3 1.5F DV** | 27 | **HYP7-Dorsal 1.5F DV** | 25 |
| **V3 1.5F AP** | 28 | **HYP7-Dorsal 1.5F AP** | 15 |
| **V6 1.5F DV** | 26 | **HYP7-Ventral 1.5F DV** | 23 |
| **V6 1.5F AP** | 25 | **HYP7-Ventral 1.5F DV** | 31 |
| **H1 1.7F DV** | 49 | **HYP7 1.7F DV** | 23 |
| **H1 1.7F AP** | 43 | **HYP7 1.7F AP** | 9 |
