# Supplementary material for: The interplay of stiffness and force anisotropies drives embryo elongation
Source: eLife. 2017 Feb 15;6:e23866. doi: 10.7554/eLife.23866 (PMC5371431; doi:10.7554/eLife.23866)
Supplement: Supplementary file 2. — DOI: http://dx.doi.org/10.7554/eLife.23866.019 [file elife-23866-supp2.docx]

| ***Initial width*** | **0.6 μm** | **0.5 μm** | **0.4 μm** | **0.3 μm** | **0.2 μm** |
| --- | --- | --- | --- | --- | --- |
| **H1 1.3F DV** | 0.150 | 0.145 | 0.14 | 0.134 | 0.129 |
| **H1 1.3F AP** | 0.231 | 0.215 | 0.2 | 0.185 | 0.173 |
| **H1 1.5F DV** | 0.216 | 0.212 | 0.207 | 0.203 | 0.198 |
| **H1 1.5F AP** | 0.214 | 0.207 | 0.201 | 0.194 | 0.189 |
| **H1 1.7F DV** | 0.253 | 0.251 | 0.249 | 0.246 | 0.244 |
| **H1 1.7F AP** | 0.201 | 0.2 | 0.197 | 0.195 | 0.193 |
| **V3 1.3F DV** | 0.347 | 0.337 | 0.326 | 0.315 | 0.303 |
| **V3 1.3F AP** | 0.061 | 0.053 | 0.046 | 0.041 | 0.038 |
| **V3 1.5F DV** | 0.348 | 0.345 | 0.34 | 0.334 | 0.328 |
| **V3 1.5F AP** | 0.265 | 0.259 | 0.25 | 0.241 | 0.233 |
| **V6 1.3F DV** | 0.068 | 0.063 | 0.059 | 0.056 | 0.053 |
| **V6 1.3F AP** | 0.104 | 0.092 | 0.082 | 0.075 | 0.069 |
| **V6 1.5F DV** | 0.235 | 0.229 | 0.223 | 0.217 | 0.211 |
| **V6 1.5F AP** | 0.183 | 0.172 | 0.161 | 0.151 | 0.143 |
