# Supplementary material for: The interplay of stiffness and force anisotropies drives embryo elongation
Source: eLife. 2017 Feb 15;6:e23866. doi: 10.7554/eLife.23866 (PMC5371431; doi:10.7554/eLife.23866)
Supplement: Supplementary file 3. — (see Materials and methods). DOI: http://dx.doi.org/10.7554/eLife.23866.020 [file elife-23866-supp3.docx]

| **Name** | ***Plateau***  **(μm)** | **SEM** | **γ (1/s)** | **SEM** | **Initial recoil speed**  **(μm/s)** | **SEM** | **Half-time (s)** | **SEM** |
| --- | --- | --- | --- | --- | --- | --- | --- | --- |
| **H1 1.3F DV** | 1.092 | 0.023 | 0.380 | 0.056 | 0.187 | 0.032 | 1.823 | 0.270 |
| **H1 1.3F AP** | 1.100 | 0.028 | 0.273 | 0.038 | 0.136 | 0.022 | 2.542 | 0.350 |
| **H1 1.5F DV** | 1.406 | 0.014 | 0.470 | 0.031 | 0.379 | 0.028 | 1.476 | 0.096 |
| **H1 1.5F AP** | 1.311 | 0.012 | 0.472 | 0.030 | 0.336 | 0.024 | 1.468 | 0.093 |
| **H1 1.7F DV** | 2.419 | 0.028 | 0.475 | 0.027 | 0.864 | 0.059 | 1.459 | 0.084 |
| **H1 1.7F AP** | 1.696 | 0.018 | 0.527 | 0.035 | 0.577 | 0.044 | 1.316 | 0.087 |
| **V3 1.3F DV** | 1.295 | 0.024 | 0.302 | 0.027 | 0.210 | 0.022 | 2.298 | 0.205 |
| **V3 1.3F AP** | 0.832 | 0.029 | 0.270 | 0.081 | 0.063 | 0.021 | 2.570 | 0.772 |
| **V3 1.5F DV** | 1.460 | 0.036 | 0.267 | 0.027 | 0.230 | 0.029 | 2.592 | 0.265 |
| **V3 1.5F AP** | 1.240 | 0.033 | 0.258 | 0.032 | 0.165 | 0.025 | 2.689 | 0.329 |
| **V6 1.3F DV** | 1.015 | 0.022 | 0.483 | 0.098 | 0.201 | 0.045 | 1.434 | 0.290 |
| **V6 1.3F AP** | 0.874 | 0.015 | 0.402 | 0.074 | 0.110 | 0.022 | 1.726 | 0.316 |
| **V6 1.5F DV** | 1.409 | 0.020 | 0.429 | 0.037 | 0.347 | 0.035 | 1.615 | 0.140 |
| **V6 1.5F AP** | 1.248 | 0.020 | 0.399 | 0.041 | 0.259 | 0.031 | 1.737 | 0.178 |
